# Severity of COVID-19 and Treatment Strategy for Patient With Diabetes

**DOI:** 10.3389/fendo.2021.602735

**Published:** 2021-04-30

**Authors:** Shi Jin, Weina Hu

**Affiliations:** ^1^ Department of Endocrinology, The Fourth Affiliated Hospital of China Medical University, Shenyang, China; ^2^ Department of Cardiology, The Fourth Affiliated Hospital of China Medical University, Shenyang, China

**Keywords:** Diabetes mellitus, COVID-19, SARS-CoV-2, cytokine storm, glycemic control

## Abstract

Coronavirus disease 2019 (COVID-19), which was named by the World Health Organization (WHO) in February 2020, has quickly spread to more than 200 countries around the world and was declared as a global pandemic in March 2020. The severity of the disease makes it more prone to severe symptoms and higher mortality rates in patients, especially those who are with comorbidities, including high blood pressure, cardiovascular disease, obesity, and diabetes, increases the concern over the consequences of this pandemic. However, initial reports do not clearly describe whether diabetes itself or associated comorbidities or treatment strategies contribute to the severe prognosis of COVID-19 infections. Various clinical trials are being conducted on glucose-lowering agents but to date, there is no standard treatment protocol approved for COVID-19 cases with pre-existing diabetes. This review is aimed to decipher the potential risk factors of COVID-19 involved from existing evidence. Identification of a novel therapeutic strategy could be beneficial for combating SARS-CoV-2, which might be dreadful to debilitating people who have diabetes.

## Introduction

Severe acute respiratory syndrome coronavirus 2 (SARS-CoV-2), the cause for coronavirus disease 2019 (COVID-19), has infected more than 126 million people in the world and over 2.76 million deaths have been reported worldwide at the time this review was written (1). This virus is a new enveloped beta-coronavirus with a single stand that shares 82% of genomic similarities with human SARS-CoV, the virus responsible for the SARS pandemic in 2003 (2). SARS-CoV-2 has a higher reproduction rate compared to other beta-coronaviruses such as SARS-CoV and Middle East Respiratory Syndrome (MERS-CoV), indicating a greater risk for global health (3). The COVID-19 outbreak has caused a much higher number of deaths (2.76 million total deaths until March 28th, 2021) than the other coronavirus respiratory syndromes (8096 cases with 774 total deaths for 2003 SARS outbreak while the 2012 MERS outbreak has 2519 confirmed cases with 866 total deaths) (1, 4, 5). As the number of confirmed cases and death increasing exponentially, using epidemiological data to characterize COVID-19 patients could help to control the spread and also for the development of interventions for the disease. The characteristic of a respiratory virus is multi-organ damage and lungs, heart and kidneys are often the major affected organs.

Studies found that having one or more comorbidities is linked to the increased severity of COVID-19. In a systematic review and meta-analysis with a total number of 46248 confirmed cases, data showed that hypertension and diabetes are the most common comorbidities among COVID-19 patients (6). Generally, personal history of diabetes or newly diagnosed diabetes was ascertained in medical records or a self-reported diagnosis with the defined diagnostic criteria according to the WHO diagnostic criteria: fasting plasma glucose ≥ 7.0 mmol/L (≥126 mg/dL) or 2-h plasma glucose ≥ 11.1 mmol/L (≥ 200 mg/dL) or HbA1c ≥ 48 mmol/mol (6.5%) (7, 8). Studies also found that older age, hypertension, obesity, diabetes, cardiovascular disease, and chronic obstructive pulmonary disease are most commonly observed in patients with severe COVID-19 and those who died (9, 10). People with diabetes have a higher risk of viral infection in previous respiratory disease (11). Although diabetes does not seem to increase the risk for COVID-19 in some regions in Europe such as Italy (12), the risk for COVID-19 associated with diabetes is increased in most parts of the world as well as the mortality rates.

However, whether diabetes per se or together with the concomitant comorbidities contribute to the worse prognosis remains to be fully uncovered. Therefore, this review aims to highlight potential risk factors in patients with COVID-19 comorbid with diabetes. We will also discuss the specific therapeutic strategies being used for people who have both COVID-19 and diabetes.

## Potential Risk Factors of COVID-19

### Age and Gender

COVID-19 morbidity and mortality are higher in older and male individuals. In a systematic literature review and meta-analysis of 13 studies including a total number of 3027 COVID-19 patients and in aged patients over 65, Zheng et al. reported a greater risk of mortality and more comorbidities such as hypertension, diabetes also greatly affecting the prognosis of the COVID-19 (13). Data from an Israeli study of 5769 recovered patients showed that younger individuals, not only are less likely to have severe COVID-19 requiring ICU and hospitalization but have an average faster recovery rate from SARS-CoV-2 infection (14). In a large Chinese case study of a total of 72314 patients, the fatality rate of the overall case is 2.3% but it was increased by up to 14.8% in patients aged 80 and older (15). The prevalence of diabetes is increased with age both in the general population and in patients with COVID-19. Patients with COVID-19 and diabetes have an older average age than those without diabetes. In one retrospective study involving 904 patients with COVID-19 (136 with diabetes), patients with diabetes were at least more than 10 years older compared to that of non-diabetic patients (7). Another study including a matched population of patients with and without diabetes found that survivors were much younger than non-survivors and older age (more than 70 years) is an independent predictor of severity of COVID-19 such as in-hospital death (16).

A preliminary study showed an overall even distribution of SARS-CoV-2 infections between men and women (51% versus 47%, respectively) (17). However, the fatality rates are 2-fold higher in males than females (18). What’s more, the sex distribution of recovering cases was 36% and 65% for males and females, respectively (19). Another study concluded that male and age serve as risk factors for poorer outcomes in COVID-19. There was a 12-fold higher risk in patients aged 80 years and older than those 50-59 years old and men have twice the risk as women (20). In a recent risk assessment for the COVID-19 confirmed cases from European Union/European Economic Area (EU/EEA) countries and the United Kingdom (UK), data described that the male-to-female ratio overall was 0.9, with more males than females admitted to hospital, requiring intensive care or respiratory support and also dying (21).

### High Blood Pressure

High blood pressure (hypertension) is the most common comorbidity in severe COVID-19 patients (22). Studies speculated that SARS-CoV-2 might directly bind to angiotensin-converting enzyme 2 (ACE2) to enter target cells (23). ACE2 is widely expressed in the upper airways, lung, heart, liver, kidney, ileum, testis and brain and it plays an important role in anti-inflammatory responses (24–26). Recent studies have investigated the link between disease severity in COVID-19 patients with hypertension and their medical therapy. In a clinical trial of 3017 hospitalized COVID-19 patients, data showed that 53% were hypertensive. Besides, the mortality rates among patients on angiotensin-converting enzyme inhibitors (ACEI) and angiotensin receptor blockers (ARBs) treatment were lower compared to other anti-hypertensive drugs. These results were supported by another Chinese study that there was a lower rate of severe diseases and a lower rate of the inflammatory response in COVID-19 patients treated with ACEI and ARBs versus patients treated with other antihypertensive agents (27). However, some *in vitro* and animal studies have demonstrated that ACEI and ARBs increased the expression of ACE2, and those drugs could facilitate the infection of target organs and exacerbate COVID-19 disease progression (28, 29). Moreover, the detailed relationship between ACEI and the renin-angiotensin system (RAS) in humans is not yet clearly understood (30). In recent systematic reviews and meta-analysis studies, data showed that hypertension is one of the major comorbidities in COVID-19 fatal cases (31) and COVID-19 patients with hypertension have a significantly increased mortality risk (32) and also a higher risk of ICU admission (33).

### Cardiovascular Disease

A high prevalence of the cardiovascular disease has been observed in patients with COVID-19 (34) and clinical studies have also reported the association between COVID-19 and cardiovascular disease (35). Pre-existing cardiovascular disease was believed to be linked with poorer outcomes and increased risk of death in COVID-19 patients. Other studies have also observed higher troponin concentration in patients with more severe COVID-19 (36). Although the predominant clinical symptoms of COVID-19 is viral pneumonia (37–39), COVID-19 can also cause cardiovascular complications such as myocardial injury, myocarditis, acute coronary syndrome (ACS), heart failure, arrhythmias, sudden cardiac arrest, coagulation abnormalities and thrombosis (40–43). A case study in South Korea reported that a COVID-19 patient was diagnosed as acute myocarditis and this patient presented very high levels of cardiac troponin I and N-terminal (NT) pro B-type natriuretic peptide (NTproBNP), the two most sensitive clinical biomarkers for myocardial injury (44), although no SRAS-Cov-2 genomes from myocardial biopsy samples were observed (45).

Recently, several cases of stress-induced cardiomyopathy with COVID-19 have been reported, while the past medical histories of these patients were unremarkable (45–47). The postulated underlying mechanism is that COVID-19 pneumonia decreased systemic oxygenation supply and while it inversely increased cardiac demand, immune dysregulation and electrolyte imbalance (48).

In summary, these findings suggest that cardiovascular disease is not only a common symptom of COVID-19 but also a risk factor for poor prognosis. Although we do not understand the mechanisms underlying COVID-19-related cardiovascular disease, this could be largely attributable to systemic inflammation based on the available clinical findings.

### Obesity

Obesity is now being recognized as a risk factor for severe outcomes and the death of COVID-19. Evidence from around the world suggesting obese people are at greater risk of becoming seriously ill from COVID-19, means there must be an extra reason to take on obesity (49, 50). Importantly, a study of 4103 patients with COVID‐19 disease in New York reported that the most important clinical features leading to hospital admission were aged (over 65 years) and obesity itself, more than hypertension, cardiovascular disease or diabetes (51). In this study, body mass index (BMI, >40 kg/m^2^) is one of the strongest hospitalization risks for COVID-19 positive patients. This finding was consistent with the results from the CORONADO study. It was found that in people with diabetes hospitalized for COVID-19, BMI (>40 kg/m^2^) was independently associated with the severity of COVID-19 but not long-term glycemic control assessed by HbA1c (8). Multiple underlying mechanisms are accounted for this association. Firstly, the alteration of respiratory performance and impaired lung perfusion may be due to abdominal fat (52) and intravascular disseminated coagulation (53). Secondly, pre-existing comorbidities such as hypertension and diabetes are prothrombotic conditions (54, 55) that contribute to worse prognosis in COVID-19 patients. This was supported by a Germany autopsy study that found that deep venous thromboembolism was observed in 7 of 12 patients and pulmonary embolism was the direct cause of death for 4 patients (56). Finally, obesity is often linked with inadequate and excessive immunological responses and chronic inflammation which could rapidly mediate disease progression to multi-organ failure in severe COVID-19 patients (57).

Furthermore, another explanation is based on the findings that SARS-CoV-2 has a high affinity for human ACE2. And ACE2 is much highly expressed in adipose tissue compared to that in the lung, major SARS-CoV-2 target tissue and obese individuals have more adipose tissue, therefore an increased ACE2‐expressing cell numbers and consequently a larger amount of ACE2 (58). ACE2 was believed to be the receptor for the entry of SARS-CoV-2 into host cells (59).

### Inflammation

Higher levels of inflammatory markers in the blood (such as C-reactive protein and ferritin), an increased neutrophil-to-lymphocyte ratio and increased serum levels of inflammatory cytokines and chemokines have been associated with COVID-19 disease severity and death (51, 60). The cytokine profiles in severe COVID-19 patients are similar to those in cytokine release syndromes, with increased production of cytokines such as interleukin (IL)-6, IL-7 and tumor necrosis factor (TNF) and also CXC-chemokine ligand 10 (CXCL10) (61). Studies have reported that inflammatory infiltration was observed in the lung, heart, kidney, spleen, and lymph nodes (62–64). Several reports have also observed higher concentrations of C-reactive protein, IL-6, IL-10, ferritin, leukocytes and lower lymphocyte percentage in severe COVID-19 patients compared to that of non-severe patients (65, 66). A low-grade systemic and chronic inflammation was often observed in diabetes. Thus, a dysregulated inflammatory innate and an adaptive impaired immune response consequently occur in diabetic patients. Therefore, a more severe disease in COVID-19 patients with diabetes may be the result of a cytokine storm, in which the patient’s immune system fights against SARS-CoV-2 and inflicts compromised damage on its organs. Several studies have reported that COVID-19 patients with diabetes have higher lymphopenia incidence and increased proinflammatory biomarkers than those without diabetes (67, 68). Moreover, increased inflammation such as elevated IL-6 and TNF levels were also observed in obese patients, thus favors COVID-19 disease progression, and worsens the lung and heart functions (69). Therefore, obesity is also an important predisposing factor for this phenomenon.

### Hyperglycemia-the Importance of Glycemic Control

Diabetes is characterized by impaired glucose homeostasis resulting from insulin resistance or deficiency. As we know, diabetes is a well-established risk factor and predictor for elevated morbidity and mortality in various diseases such as cardiovascular diseases, cancer, and infection diseases (70–73).

In a cohort of 1561 patients with COVID-19 in two hospitals from Wuhan, those with diabetes are more likely to require an intensive care unit (ICU) admission or to die (16). In a centered, retrospective study, Yang et al. demonstrated that among cases with a poor outcome, in a group of 32 non-survivors of 52 ICU patients, 7 (22%) had diabetes (74). Data from a British hospital including 10926 COVID-19 related death in a cohort of 17278392 adults showed that the risk of death is much higher in those with uncontrolled diabetes (20). Other studies also described that there was a worse outcome in COVID-19 patients is diabetes (75), therefore, result in more hospitalizations, more admission for ICU and more death (33).

As we know, the virulence of some pathogens is increased during the hyperglycemic environment. It was reported that phagocytosis and chemotaxis are impaired and the production of T cells and neutrophils in response to infection is also reduced in diabetes (76). Generally, the immune response is damaged in diabetic COVID-19 patients with poor blood glucose control. Among the COVID-19 patients with diabetes, higher incidences of neutrophilia and lymphopenia are observed in those with higher levels of blood glucose concentration (67). Recently, several studies have shown that impaired pulmonary function significantly correlates with blood glucose levels (77, 78). Therefore, the vulnerability to respiratory infections is increased along with reduced pulmonary capacity. This might be another factor that diabetes is more commonly observed in severe COVID-19 patients. Moreover, in a total of 663 COVID-19 patients’ study, type 2 diabetes was found to be associated with no improvement in patients with COVID-19 and type 2 diabetes patients were prone to developing into severe and critical condition of COVID-19 and having a poorer therapeutic effect. Furthermore, having a severe and critical condition and decreased lymphocyte count were independent risk factors associated with poor therapeutic effects in COVID-19 patients with type 2 diabetes (79). Interestingly, in a population-based cohort study with diagnosed type 1 and type 2 diabetes in England, Holman found that increased COVID-19-related mortality was associated not only with cardiovascular and renal complications of both types of diabetes but also independently with glycemic control and BMI (80).

A recent study reported that COVID-19 patients with diabetes and uncontrolled hyperglycemia (defined as two or more blood glucose > 180 mg/dL within any 24-hour period with an HbA1C < 6.5% or no HbA1C testing during hospitalization) were associated with longer hospitalization and higher mortality (81). Studies also found that higher blood glucose concentration at admission was associated with the poorer primary outcome. In a retrospective analysis of 85 COVID-19 patients, Iacobellis et al. reported that hyperglycemia on the first day of admission is the best predictor of radiographic imaging, regardless of pre-existing diabetes (82). Furthermore, hyperglycemia during treatment was a risk factor for death in patients with severe COVID-19 (83). In a retrospective multi-center study including COVID-19 patients hospitalized in Spain, it was found that hyperglycaemia (>180 mg/dL) is a strong predictor of all-cause mortality in non-critically hospitalized COVID-19 patients regardless of diabetes history (84). Thus, glucose testing and glycemic control are important to all COVID-19 patients even without pre-existing diabetes, as most COVID-19 patients are prone to glucose metabolic disorders. This is because the ACE receptors where SARS-CoV-2 binds to enter target cells are also expressed in pancreatic β cells (24). This could induce acute impairment of insulin secretion and β cell destruction resulting in *de novo* diabetes development.

In summary, uncontrolled glycemic at admission and hospitalization exacerbate poor outcomes of COVID-19 patients. In COVID-19 patients with hyperglycemia, therapeutic strategies combined with glycemic control should be considered to reduce the risk of severe outcomes and mortality.

## Therapeutics-Treatment Specific to Patients Having Both COVID-19 and Diabetes

Medical teams should ensure sufficient glycemic control in COVID-19 patients with diabetes. This requires full consideration of all potential complications the therapies may generate which will be used for those patients.

Insulin treatment is a general therapy for both types of diabetes. However, insulin therapy should be decided based on the severity of COVID-19 and those patients should be intensely monitored, although this treatment has been recommended in severe COVID-19 patients with diabetes (85). One study found that poorer clinical outcomes in patients treated with insulin compared with those under metformin treatment (7). Despite better outcomes reported in diabetic patients with COVID-19 on metformin, this drug should be discontinued if patients with respiratory distress, renal dysfunction, or heart failure due to acidosis (85). In the CORONADO study, Cariou et al. reported that the use of metformin was lower in patients who died and other therapies such as insulin treatment, renin–angiotensin–aldosterone system (RAAS) blockers, β-blockers and loop diuretics were associated with death on day 7. They believed this finding could be attributed to the underling comorbidities and diabetic complications in people who died because these patients were received more frequent treatment including insulin and other multiples drugs (8). A recent study reported significantly higher postprandial glycemic fluctuations and exposure to hyperglycemia were observed among patients with COVID-19 followed by continuous glucose monitoring (86). Thus, continuous blood glucose monitoring should also be included during the treatment process.

Sodium-glucose transporter 2 inhibitors should also be treated with caution due to their adverse effects such as ketoacidosis and impaired fat metabolism (87). Besides, Care should also be taken with the use of glucagon-like receptor-1 (GLP-1R) analogues since they may cause diarrhea, nausea, vomiting and headaches (88). In a recent multicenter, case-control, retrospective, and observational study, sitagliptin, an oral and highly selective dipeptidyl peptidase 4 (DPP4) inhibitor, was used as an add-on therapy to the standard of care in patients with type 2 diabetes and COVID-19. Sitagliptin treatment was found to be associated with reduced mortality, an improved clinical outcome and a greater number of hospital discharges in this study (89). These beneficial effects may be attributed to the shared disease pathophysiology pathways in coronavirus infections and type 2 diabetes. DPP4 and ACE2, two major coronavirus receptor proteins, are well-established transducers of metabolic signals and pathways regulating inflammation, cardiorenal physiology, and glucose homeostasis. Moreover, glucose-lowering drugs such as the DPP4 inhibitors, widely used in type 2 diabetes patients, are known to modify the biological activities of multiple immunomodulatory substrates (90).

As we know, it is a multistep process for virus infection. Researchers have proposed several targets to treat COVID-19. The beneficial effects of ACEI and ARBs for kidney and heart in diabetes have already been proven (91). However, as stated above, for COVID-19 patients with diabetes, the use of ACEI and ARBs for those patients should be carefully discussed based on the context of the individuals.

Glucocorticoids are known to cause hyperglycemia in patients with or without pre-existing diabetes. However, it has been used for the treatment of severely ill patients to suppress the very high levels of cytokines and c-reactive peptides which are often observed in those patients, although they can exacerbate insulin resistance, reduce insulin sensitivity and cause severe hyperglycemia. No studies have found that they could decrease mortality or slow viral clearance in clinical.

## Conclusions

Patients with COVID-19 and diabetes are at greater risk for more severe infections, poorer prognosis and much higher mortality compared to those patients without diabetes. The grave prognosis and the risk factors for patients with diabetes are well linked with older age, sex, high blood pressure, cardiovascular disease, obesity, inflammation and hyperglycemia. All those factors contribute to the increased risk of getting severely ill in those individuals. It is a great challenge for blood glucose management in COVID-19 patients because it requires more detailed strategies for medical team integration and fully considerations of all possible complications and death.

It is also clear that the relationship between diabetes and COVID-19 is tightly linked together and it requires more research to fully uncover the specific mechanisms of SARS-CoV-2 such as how SARS-CoV-2 impairs the pancreatic islets, deteriorates insulin homeostasis and induce *de novo* diabetes development.

## Author Contributions

SJ and WH wrote different sections of the manuscript. WH revised, wrote, and prepared the manuscript. All authors contributed to the article and approved the submitted version.

## Funding

This work was supported by the Department of Science and Technology of Liaoning province (2019-ZD-0774).

## Conflict of Interest

The authors declare that the research was conducted in the absence of any commercial or financial relationships that could be construed as a potential conflict of interest.
